# Glucose Metabolism Reprogramming of Primary Tumor and the Liver Is Associated With Disease-Free Survival in Patients With Early NSCLC

**DOI:** 10.3389/fonc.2021.752036

**Published:** 2021-10-28

**Authors:** Hongpei Tan, Mengtian Ma, Jing Huang, Wenhao Zhu, Shuo Hu, Kai Zheng, Pengfei Rong

**Affiliations:** ^1^ Department of Radiology, Third Xiangya Hospital, Central South University, Changsha, China; ^2^ Department of Anesthesiology, Zhuzhou Central Hospital, Zhuzhou, China; ^3^ Xiangya School of Medicine, Central South University, Changsha, China; ^4^ Department of Nuclear Medicine, Xiangya Hospital, Central South University, Changsha, China; ^5^ PET/CT Center, Hunan Cancer Hospital, Changsha, China; ^6^ The Affifiliated Cancer Hospital of Xiangya School of Medicine, Central South University, Changsha, China

**Keywords:** NSCLC, FDG (18F-fluorodeoxyglucose)-PET/CT, metabolism reprogramming, DFS = disease-free survival, Liver glucose metabolism, splenic glucose metabolism

## Abstract

**Purpose:**

Tumor promote disease progression by reprogramming their metabolism and that of distal organs, so it is of great clinical significance to study the changes in glucose metabolism at different tumor stages and their effect on glucose metabolism in other organs.

**Methods:**

A retrospective single-centre study was conducted on 253 NSCLC (non-small cell lung cancer) patients with negative lymph nodes and no distant metastasis. According to the AJCC criteria, the patients were divided into different groups based on tumor size: stage IA, less than 3 cm (group 1, n = 121); stage IB, greater than 3-4 cm (group 2, n = 64); stage IIA, greater than 4-5 cm (group 3, n = 36); and stage IIB, greater than 5-7 cm (group 4, n = 32). All of the patients underwent baseline ^18^F-FDG PET/CT scans, and the primary lesion SUVmax (maximum standardized uptake value), liver SUVmean (mean standardized uptake value), spleen SUVmean, TLR (Tumor-to-liver SUV ratio) and TSR (Tumor-to-spleen SUV ratio) were included in the study, combined with clinical examination indicators to evaluate DFS (disease free survival).

**Results:**

In NSCLC patients, with the increase in the maximum diameter of the tumor, the SUVmax of the primary lesion gradually increased, and the SUVmean of the liver gradually decreased. The primary lesion SUVmax, liver SUVmean, TLR and TSR were related to disease recurrence or death. The best predictive parameters were different when the tumor size differed. SUVmax had the highest efficiency when the tumor size was less than 4 cm (AUC:0.707 (95% CI, 0.430-0.984) tumor size < 3 cm), (AUC:0.726 (95% CI, 0.539-0.912) tumor size 3-4 cm), liver SUVmean had the highest efficiency when the tumor size was 4-5 cm (AUC:0.712 (95% CI, 0.535-0.889)), and TLR had the highest efficiency when the tumor size was 5-7 cm [AUC:0.925 (95%CI, 0.820-1.000)].

**Conclusions:**

In patients with early NSCLC, glucose metabolism reprogramming occurs in the primary lesion and liver. With the increase in tumor size, different metabolic parameters should be selected to evaluate the prognosis of patients.

## Introduction

Lung cancer is an important global health problem. In 2020, more than 1.8 million people worldwide died of lung cancer. Non-small cell lung cancer (NSCLC) is particularly aggressive, and even patients with early NSCLC are at risk of death (1). In clinical work, tumor glucose metabolism has been a very important index for clinical evaluation of the progression and prognosis of NSCLC (2).

Metabolic changes in tumor occur at all stages of tumorigenesis and development. Tumor has increased energy uptake, which further increases proliferation and invasion through metabolic reprogramming (3, 4), Metabolic reprogramming is also associated with immune escape (5, 6), studies have shown that tumor can also promote tumor development by regulating the metabolism of tumor-infiltrating immune cells (7). Therefore, Metabolic changes in tumor regions are very important for tumor research. The metabolic regulation of tumor is not limited to the scope of tumor, related studies have mentioned that tumor can affect the metabolic level of other organs (8, 9). This finding suggests that the metabolic level of both the primary tumor and other organs will change dynamically with the progression of the tumor. It also suggests that we should analyse the tumor in the general environment of the whole body. Therefore, how to accurately use glucose metabolism to evaluate tumor prognosis at different stages needs further research. A single metabolic index may not be able to predict the prognosis of patients at each stage of cancer, and stratified analysis of tumor patients should be conducted to obtain the best prognostic index at each stage. Among the many organs, the liver and spleen are important for the inflammatory response and tumor immunity. Therefore, our aim was to explore the metabolic changes in tumor at different stages and their effects on other organs and to determine the effects of these metabolic changes on prognosis. In this paper, we retrospectively studied the changes in the metabolism of tumor of different sizes and their effects on the metabolism of the liver and spleen in NSCLC. Metabolic changes were further combined with clinical indicators to investigate the risk factors affecting disease-free survival (DFS).

## Methods

### Patients

**Figure 1:**
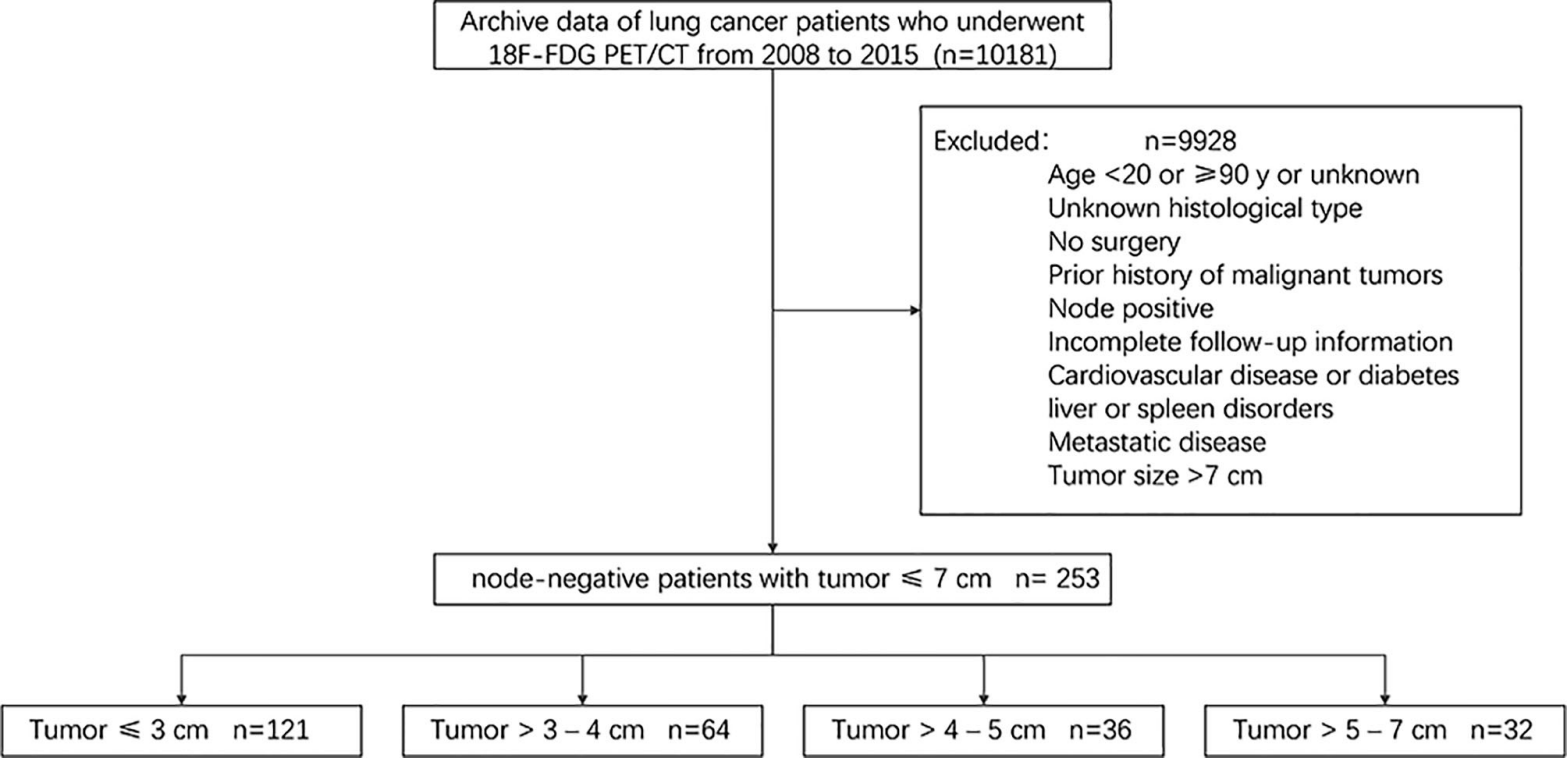
Flow diagram shows the details of patient exclusion.

In this retrospective single-centre study, the following inclusion/exclusion criteria were used to select patients from the institutional database. Since studies have shown that positive lymph nodes can affect the survival of patients with NSCLC, we did not include patients with positive lymph nodes (10, 11). The inclusion criteria were as follows: a) age greater than 18 years and younger than 90 years; b) surgical intervention for lung lesions between 2008 and 2015; and c) fluorine-18-fluorodeoxyglucose positron emission tomography/computed tomography (^18^F-FDG PET/CT) scan performed to identify lung lesions in our institution within 45 days before surgery. The exclusion criteria were as follows: a) histology other than lung adenocarcinoma or squamous cell carcinoma; b) associated with other cancer types or previous cancer; c) local lymph node positivity or distant metastasis; d) tumor larger than 7 cm; e) Patients with other medical conditions, such as cardiovascular disease, diabetes or liver or spleen disorders; and f) incomplete follow-up information. A total of 10181 patients were selected from the institutional database, and the above inclusion and exclusion criteria were applied to select a cohort of 253 patients. A brief flow diagram is shown in **Figure 1**. For all patients, available clinical parameters, such as age, sex, coagulation function, hematologic parameters, histological type, and tumor grade, were recorded. The analysis did not consider smoking habits or performance status. Histology and/or imaging was used to identify the presence of distant metastases and whether lymph nodes were positive. At the time of hospitalization, all patients had signed the relevant consent that the data could be used in clinical research.

According to the current 8th edition of the American Joint Committee on Cancer (AJCC) cancer staging manual (12), the patients were further subdivided as follows: stage IA, less than 3 cm (group 1, n = 121); stage IB, greater than 3-4 cm (group 2, n = 64); stage IIA, greater than 4-5 cm (group 3, n = 36); and stage IIB, greater than 5-7 cm (group 4, n = 32).

The endpoint of this study was DFS, which was defined as the time between the date of surgery and the date of recurrence (event), which refers to tumor recurrence or tumor-related death, or the date of the last visit (after examination). The follow-up period was five years, and recurrence or death within five years was defined as the occurrence of an event. The study was approved by the institutional ethics committee.

### ^18^F-FDG PET/CT Image Acquisition

^18^F-FDG PET/CT image acquisition was carried out according to version 1.0 of the European Association of Nuclear Medicine (EANM) guidelines on an integrated PET/CT scanner (General Electric Discovery ST8, General Electric Healthcare, Chicago, IL). In short, proper patient preparation (at least 6 hours of fasting) and adequate blood glucose levels (<110 mg/dL) were required. Images were obtained 60 ± 5 minutes after the intravenous injection of 370 MBq/kg of ^18^F-FDG. First, a low-dose CT scan without contrast enhancement (120 mA, 150 kV, 512 × 512 matrix, the pitch of 1.75, reconstruction thickness and interval of 3.75 mm) for a precise anatomical localization and attenuation correction was performed. Next, a three-dimensional PET scan (thickness of 3.27 mm) was performed from the skull base to the proximal thighs with an acquisition time of 3 min per bed position.

The PET data sets were iteratively reconstructed using an ordered-subset expectation maximization (OSEM) algorithm with attenuation correction. All collected images were displayed on the GE Healthcare Xeleris 3.0 to reconstruct the PET, CT, and PET/CT fusion images.

### Image Analysis

Two experienced senior nuclear medicine physicians who did not know the clinical information of the patients checked the PET/CT images separately, they all had more than five years of PET/CT diagnostic experience. If the results differed, they discussed the findings and then reached a consensus. According to the PET/CT images, contrast-enhanced CT or magnetic resonance imaging (MRI) was performed when necessary. The region of interest (ROI) for each patient was delineated initially around the tumor outline for the largest cross-sectional area of the primary lung lesion on both the CT and PET images, tumor size was determined at largest cross section. The maximum standardized uptake value (SUVmax) of the primary tumor was measured by carefully placing the ROI on different cross sections of the primary tumor. The mean standardized uptake value (SUVmean) of the liver was measured by selecting a region of interest (ROI) on the axial image. We used the largest cross section of the right lobe of the liver to select a relatively uniform region in the parenchyma of the liver and drew a circle with a diameter not less than 3 cm as the ROI. The liver SUVmean of each patient was measured three times, and the average value was calculated to reduce the selection deviation (13). Subsequently, in the same way, the largest cross section of the spleen was selected to draw the ROI in a relatively uniform parenchymal area. Three measurements were taken for each patient, and the average value of the three results was taken to obtain the spleen SUVmean (14). The tumor-to-liver ratio (TLR) was obtained by the ratio of SUVmax to liver SUVmean, and tumor-to-spleen ratio (TSR) was obtained by the ratio of SUVmax to spleen SUVmean (15). Tumor size was defined as the largest size of the primary tumor. According to the eighth edition of the AJCC TNM staging system, regional lymph nodes and distant metastasis were evaluated.

### Statistical Analysis

All continuous data are expressed as the mean ± standard deviation, and categorical variables are shown as numbers (percentages). The Kruskal-wallis test was used for continuous variables in the clinical and imaging features, and the Pearson chi-square test or Fisher’s exact test was used for categorical variables as appropriate. Univariate analysis for DFS was performed by the Kaplan-Meier method. The log-rank test and Cox regression analysis were used to identify factors significantly associated with DFS. Time-dependent receiver operating characteristic (timeROC) curve analysis was performed to determine the optimal cut-off and area under the curve (AUC) of primary tumor SUVmax, liver SUVmean, spleen SUVmean, TLR and TSR. Statistical analyses were performed using R (version 4.0.3, http://www.r-project.org) software. All P values less than 0.05 were considered statistically significant.

## Results

### Metabolic Changes in Tumor at Different Stages and Their Effect on the Metabolism of Other Organs

**Figure 2:**
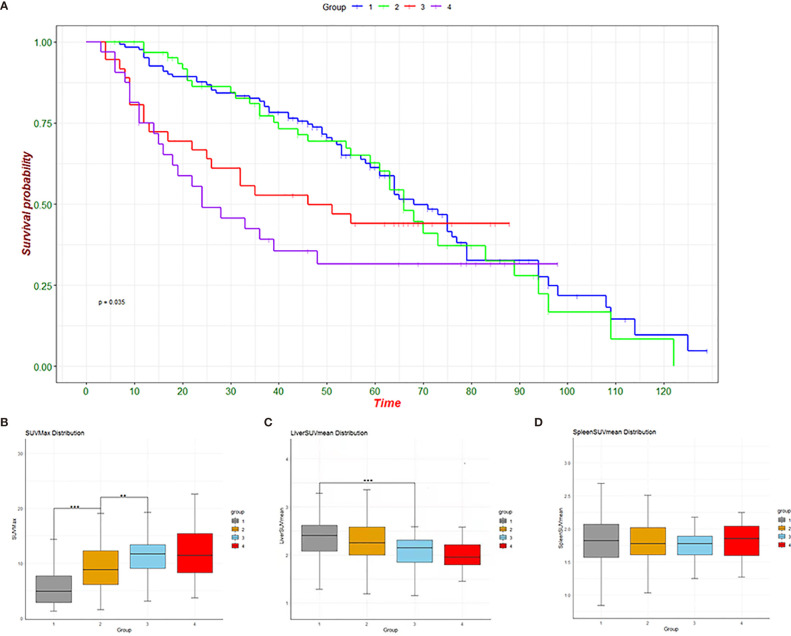
Survival graphs of the four groups **(A)** and change trend graphs of SUVmax **(B)**, liver SUVmean **(C)** and spleen SUVmean **(D)**. **p < 0.01, ***p < 0.001.

We first compared the DFS of the four groups of patients and found that there were significant differences in survival among the four groups (P<0.05) (**Figure 2A**). Further observation of the metabolic changes of patients at different stages showed that SUVmax in the primary tumor gradually increased with the progression of the tumor (**Figure 2B**), and there were significant differences between groups (1 vs 2, P<0.05; 2 vs 3, P<0.05). Liver metabolism gradually decreased with the progression of the tumor (**Figure 2C**), and there was a significant difference between the first group and the third group (1 vs 3, P<0.01). For the spleen, different tumor stages had no significant effect on glucose metabolism (**Figure 2D**).

### Indicators Related to Disease Recurrence and DFS

We first analysed patients with events (death or recurrence) and DFS (disease free survival) and found that there were differences in sex, height, weight, degree of pathological differentiation, fibrinogen (Fib) and thrombin time (TT) between the two groups. In terms of imaging metabolic indexes, there were also differences in primary lesion SUVmax, liver SUVmean, TLR and TSR between the two groups (**Table 1**).

**Table 1:** Comparison of the basic characteristics of patients with an event and patients with disease-free survival.

Characteristic (mean ± SD)	All patients (n=253)	DFS (n=110)	Events (n=143)	P
Sex (%)				
Female	77 (30.4)	43 (39.1)	34 (23.8)	0.013
Male	176 (69.6)	67 (60.9)	109 (76.2)	
Age	58.19 ± 9.10	58.32 (8.01)	58.09 (9.88)	0.845
Hight	163.20 ± 7.41	161.69 ± 7.51	164.36 ± 7.14	0.004
Weight	61.34 ± 10.74	59.49 ± 9.77	62.77 ± 11.26	0.016
SUVmax	8.37 ± 4.96	6.26 ± 4.66	9.98 ± 4.57	<0.001
Liver SUVmean	2.23 ± 0.47	2.30 ± 0.47	2.17 ± 0.45	0.027
Spleen SUVmean	1.81 ± 0.39	1.80 ± 0.39	1.82 ± 0.39	0.559
TLR	4.01 ± 2.67	2.79 ± 2.21	4.99 ± 2.78	<0.001
TSR	4.78 ± 2.85	3.64 ± 2.72	5.65 ± 2.63	<0.001
Pathology (%)				0.506
Adenocarcinoma	147 (58.1)	67 (60.9)	80 (55.9)	
SCC	106 (41.9)	43 (39.1)	63 (44.1)	
Differentiation (%)				0.002
Poor	76 (30.0)	23 (20.9)	53 (37.1)	
Moderate	115 (45.5)	50 (45.5)	65 (45.5)	
Well	62 (24.5)	37 (33.6)	25 (17.5)	
WC	6.89 ± 2.48	6.86 ± 2.87	6.91 ± 2.14	0.875
LC	1.85 ± 0.64	1.86 ± 0.61	1.85 ± 0.67	0.937
NC	4.24 ± 2.16	4.23 ± 2.35	4.25 ± 2.02	0.94
NLR	2.64 ± 2.19	2.42 ± 1.39	2.81 ± 2.64	0.156
MC	0.52 ± 0.31	0.51 ± 0.40	0.53 ± 0.21	0.537
Eos	0.22 ± 0.18	0.21 ± 0.16	0.23 ± 0.20	0.497
Bas	0.04 ± 0.02	0.04 ± 0.03	0.03 ± 0.02	0.05
Hb	132.45 ± 14.75	132.22 ± 14.51	132.64 ± 14.98	0.824
Plt	223.74 ± 72.30	227.35 ± 70.68	220.96 ± 73.65	0.487
PT	12.06 ± 6.95	11.67 ± 1.33	12.36 ± 9.17	0.439
Fib	3.89 ± 1.87	3.43 ± 1.09	4.25 ± 2.23	<0.001
APTT	33.22 ± 5.24	33.27 ± 5.26	33.18 ± 5.25	0.898
TT	16.28 ± 2.50	16.72 ± 2.25	15.95 ± 2.64	0.015

DFS, disease-free survival; TLR, tumor-to-liver standardized uptake value ratio; TSR, tumor-to-spleen standardized uptake value ratio; SCC, squamous cell carcinoma; WC, white blood cell count; LC, lymphocyte count; NC, neutrophil count; NLR, neutrophil/lymphocyte ratio; MC, monocyte count; Eos, eosinophil; Bas, basophil; Hb, hemoglobin; Plt, Platelet; PT, prothrombin time; Fib, fibrinogen; APTT, activated partial thromboplastin time; TT, thrombin time.

Using the imaging and clinical characteristics of all patients to conduct univariate survival analysis, we found that SUVmax, liver SUVmean, differentiation, Fib and so on were prognostic factors of DFS, with AUCs of 0.621 for SUVmax, 0.612 for liver SUVmean, 0.671 for TLR, 0.632 for TSR, 0.525 for pathology, 0.585 for differentiation, 0.523 for TT and 0.602 for Fib (**Table 2**). Multivariate analysis showed that the three indexes of TSR, differentiation and Fib were related to survival (**Table 3**).

**Table 2:** Univariate regression analysis of survival performed for all basic characteristics.

Characteristic		Univariate analysis		AUC
		HR (95%CI)	P	(95%CI)
Sex (%)	Female/Male	1.3 (0.9-2)	0.15	
Age	23-77	1 (0.99-1))	0.63	
Hight	145-181	1 (0.98-1)	0.61	
Weight	40-99	1 (0.99-1)	0.92	
SUVmax	1.28-31.13	1.1 (1-1.1)	<0.001	0.621 (54.19-69.92)
Liver SUVmean	0.83-4.24	0.57 (0.4-0.81)	<0.01	0.612 (53.25-69.12)
Spleen SUVmean	0.77-3.25	0.94 (0.61-1.4)	0.78	
TLR	0.48-13.50	1.2 (1.1-1.2)	<0.001	0.671 (56.69-77.44)
TSR	0.59-15.04	1.1 (1.1-1.2)	<0.001	0.632 (52.17-74.19)
Pathology	SCC/Adenocarcinoma	1 (0.75-1.5)	<0.001	0.525 (45.57-59.51)
Differentiation	Well/Moderate/Poor differentiation	0.61 (0.48-0.78)	<0.001	0.585 (51.15-65.83)
WC	2.47-23.45	1 (0.95-1.1)	0.62	
LC	0.23-4.31	0.89 (0.7-1.1)	0.36	
NC	0.00-17.69	1 (0.97-1.1)	0.32	
NLR	0.00-21.51	1 (0.98-1.1)	0.25	
MC	0.09-3.88	1.2 (0.72-1.9)	0.54	
Eos	0.00-1.29	1.7 (0.73-4.2)	0.21	
Bas	0.00-0.15	0.0024 (0.00-3.3)	0.1	
Hb	92-168	0.99 (0.98-1)	0.33	
Plt	55-438	1 (1-1)	0.18	
PT	8.90-120	1 (1-1)	0.052	
Fib	1.55-23.5	1.2 (1.1-1.3)	<0.001	0.602 (52.27-68.13)
APTT	16.80-49.4	1 (0.97-1)	0.88	
TT	4.77-26.88	0.93 (0.86-1)	0.044	0.523 (0.418-0.627)

**Table 3:** Multivariate analysis of risk factors.

Characteristic		Multivariate analysis	
		HR (95% CI)	P
TSR	0.59-15.04	1.41 (1.06-1.86)	0.018
Differentiation	Well/Moderate/Poor differentiation	0.69 (0.53-0.91)	0.009
Fib	1.55-23.5	1.14 (1.03-1.27)	0.013

### Pathological and Imaging Features Affecting DFS at Different Tumor Stages Based on Tumor Size

**Figure 3:**
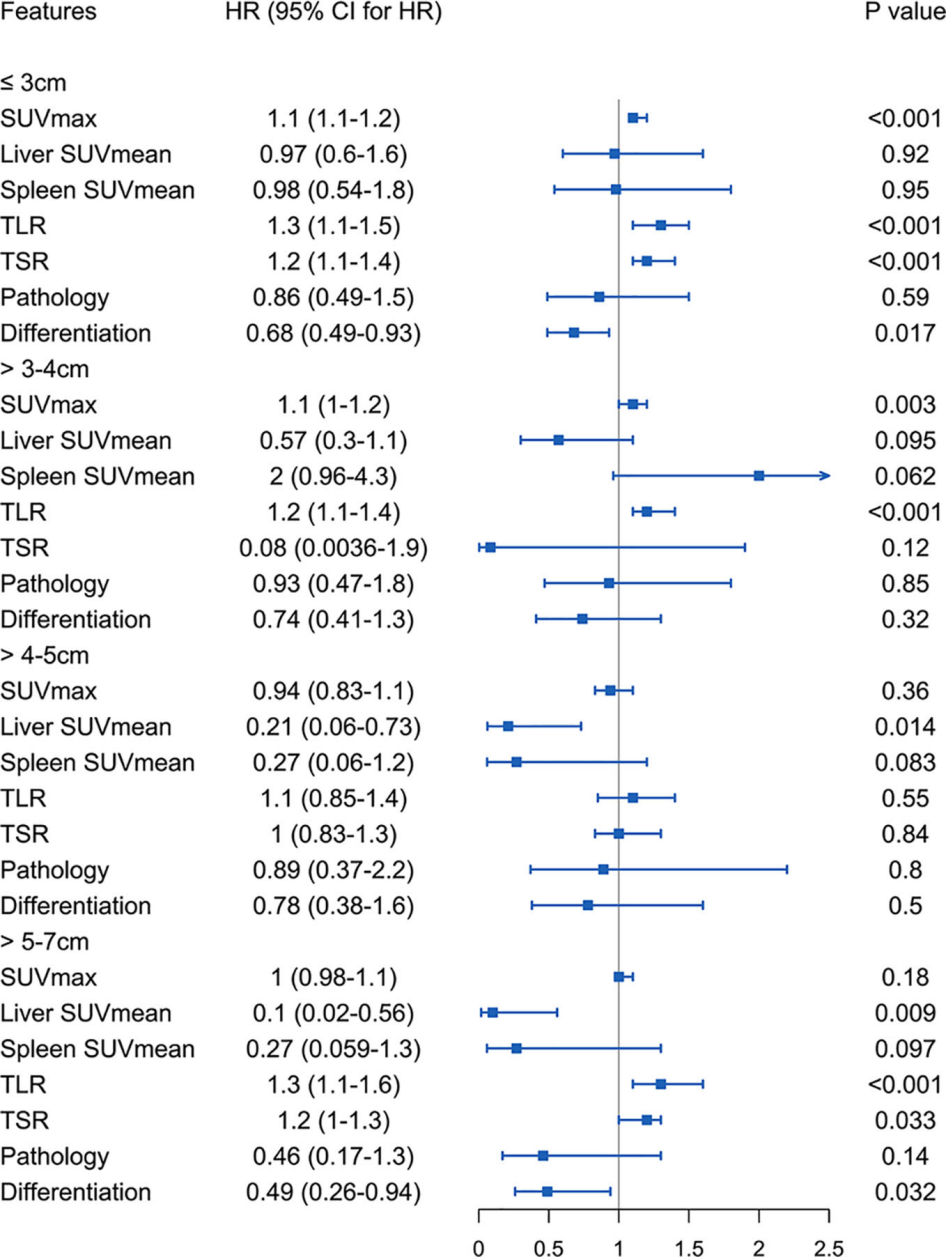
Four groups of patients stratified by tumor size using imaging metabolic indicators and pathological features to analyse the risk of survival.

We analysed the survival risk of imaging metabolic and pathological indexes in the four groups of patients (**Figure 3**). The results here show that the parameters affecting disease-free survival are different in different tumor sizes.

#### Tumor Size of 3 cm or Less

**Figure 4:**
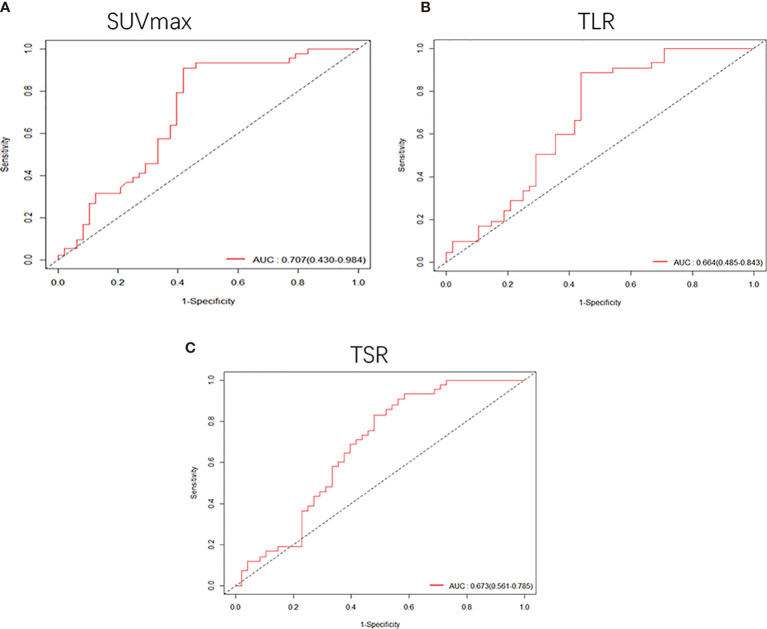
Receiver operating characteristic (ROC) curves were used to compare the SUVmax **(A)**, TLR **(B)** and TSR **(C)** of patients whose tumor size was smaller than 3 cm. The AUCs were 0.707 (95%CI, 0.430-0.984) for SUVmax, 0.664 (95%CI, 0.485-0.843) for TLR and 0.673 (95%CI, 0.561-0.785) for TSR.

Among patients with tumor less than or equal to 3 cm, SUVmax, TLR, TSR and degree of differentiation were related to the risk of DFS. A lower degree of differentiation indicated a higher risk (HR: 0.68, 95% CI, 0.49-0.93, P<0.05), and higher SUVmax (HR: 1.1, 95% CI, 1.1-1.2, P<1e-05), TLR (HR: 1.3, 95% CI, 1.1-1.5, P<4.7e-05) and TSR (HR: 1.2, 95% CI, 1.1-1.4, P<3.4e-05) were also high-risk factors. The cut-off values obtained were 4.6 for SUVmax (**Supplementary Figure S1B**), 1.92 for TLR (**Supplementary Figure S1D**) and 2.57 for TSR (**Supplementary Figure S1F**). The AUC values of these three imaging indicators were obtained by drawing ROC curves and were 0.707 (95%CI 0.430-0.984) for SUVmax, 0.664 (95%CI 0.485-0.843) for TLR, and 0.673 (95%CI 0.561-0.785) for TSR (**Figure 4**).

#### Tumor Larger Than 3 cm to 4 cm

**Figure 5:**
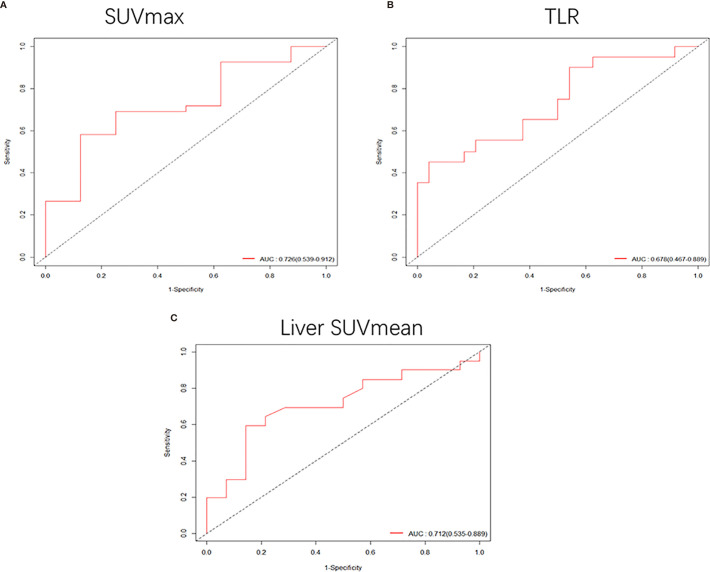
**(A, B)** When the tumor size was 3-4 cm, the receiver operating characteristic (ROC) curves based on SUVmax and TLR were 0.726 (95%CI, 0.539-0.912) and 0.678 (95%CI, 0.467-0.889), respectively. **(C)** ROC curve of liver SUVmean when the tumor size was 4-5 cm, AUC: 0.712 (95%CI, 0.535-0.889).

In the analysis of patients with a tumor size of 3-4 cm, it was found that high SUVmax (HR: 1.1, 95% CI, 1.1-1.2, P<0.001) and TLR (HR: 1.2, 95% CI, 1.1-1.4, P<0.001) were risk factors for DFS. The cut-off values obtained were 7.69 for SUVmax (**Supplementary Figure S2B**) and 3.6 for TLR (**Supplementary Figure S2D**), and the AUC values obtained by drawing ROC curves were 0.726 (95%CI, 0.539-0.912) for SUVmax (**Figure 5A**) and 0.678 (95%CI, 0.467-0.889) for TLR (**Figure 5B**).

#### Tumor Larger Than 4 cm to 5 cm

In patients with a tumor size of 4-5 cm, only low liver SUVmean was a high-risk factor for DFS (HR: 0.21, 95% CI, 0.062-0.73, P<0.014). The cut-off value we obtained was 2.15 (**Supplementary Figure S2F**), and the AUC value obtained by drawing the ROC curve was 0.712 (95%CI, 0.535-0.889) (**Figure 5C**).

#### Tumor Larger Than 5 cm to 7 cm

**Figure 6:**
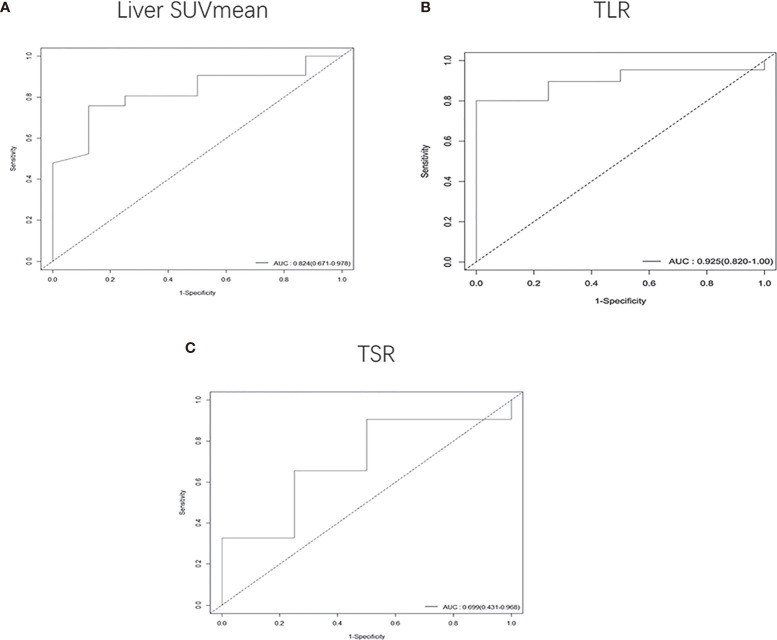
Receiver operating characteristic (ROC) curves were used to compare the liver SUVmean **(A)**, TLR **(B)** and TSR **(C)** of patients whose tumor size was 5-7 cm. The AUCs were 0.824 (95%CI, 0.671-0.978) for liver SUVmean, 0.925 (95%CI, 0.820-1.000) for TLR and 0.699 (95%CI, 0.431-0.968) for TSR.

In patients with a tumor size of 5-7 cm, we found that higher TLR (HR: 1.3, 95% CI, 1.1-1.6, P<0.001) and TSR (HR: 1.2, 95% CI, 1-1.3, P<0.05) were risk factors, and lower liver SUVmean (HR: 0.1, 95% CI, 0.018-0.56, P<0.01) and degree of differentiation (HR: 0.49, 95% CI, 0.26-0.94, P<0.05) were high-risk factors for DFS. The cut-off values ​​we obtained were 1.95 for liver SUVmean, 5.89 for TLR, and 6.32 for TSR (**Supplementary Figure S3**). The AUC values ​​obtained by drawing ROC curves were 0.824 (95%CI, 0.671-0.978) for liver SUVmean, 0.925 (95%CI, 0.820-1.000) for TLR and 0.699 (95%CI, 0.431-0.968) for TSR (**Figure 6**).

## Discussion

In this study, we evaluated the glucose metabolic changes in primary lesions and their effects on the metabolism of other organs in patients with node-negative NSCLC staged based on tumor size, and we further analysed the correlation between metabolic parameters and prognosis in terms of DFS. We found that with the increase in tumor size, the tumor had an increased glucose metabolism capacity and decreased liver metabolism, and the metabolism of both the primary lesion and the liver were related to disease recurrence. In addition, in the prognosis analysis of tumor of different sizes, we found that different metabolic parameters should be selected to evaluate the prognosis of tumor of different sizes.

^18^F-FDG uptake has been reported as a strong prognostic factor in many malignancies, including lung cancer, lymphoma, cervical cancer, and intrahepatic cholangiocarcinoma (13, 16–19). In related studies, the most commonly used organ metabolism level in addition to the primary lesion is the liver. Most studies use the liver as a metabolic reference background, and TLR can better reflect the metabolic level of the lesion area. Related research also suggests that TLR is better than SUVmax in predicting prognosis and evaluating treatment response in some tumors or at different stages of the tumor (13, 20–22). Other studies have shown that TLR can significantly distinguish high-grade and low-grade tumors (23). These studies show the importance of liver metabolism in tumor research, so in the study of tumors, researchers should not only pay attention to the primary focus itself but also investigate tumors in the general environment of the whole body.

In this study, we found that with increasing tumor size, the glucose uptake capacity gradually increased, and the DFS of patients also gradually decreased. As tumors mainly use glycolysis, the increase in glucose metabolism inevitably leads to an increase in glycolytic metabolites. A recent study showed that lactic acid, one of the products of glycolysis, can increase the function of T regulatory cells and inhibit tumor immunity, which may be one of the reasons for the poor survival of patients with higher metabolism (24). Moreover, a previous study mentioned that higher ^18^F-FDG uptake is associated with higher tumor proliferation (25). We also found that liver metabolism decreased with increasing tumor size but had no significant effect on spleen glucose metabolism. In this study, we analysed the primary tumor SUVmax, liver SUVmean, spleen SUVmean, TLR and TSR in 253 patients with NSCLC examined by ^18^F-FDG PET/CT. We first compared the differences between patients with disease recurrence or death and patients with DFS and found that there were significant differences between the two groups in primary lesion SUVmax, liver SUVmean, TLR and TSR. Univariate and multivariate survival analyses showed that the above four imaging indicators were all associated with DFS. In addition, further stratification of tumor patients according to tumor size showed that SUVmax, TLR and TSR were associated with DFS in groups with tumor sizes less than 3 cm, and SUVmax and TLR were also associated with DFS in patients with tumor sizes of 3-4 cm. This finding was consistent with that of a previous study, which mentioned that ^18^F-FDG PET had a prognostic cut-off for lung adenocarcinoma in stage IA (2). For patients with a tumor size of 4-5 cm, liver SUVmean was associated with prognosis, and for patients with a tumor size of 5-7 cm, liver SUVmean, TLR and TSR were associated with DFS. Our study shows that primary SUVmax, liver SUVmean, TLR and TSR have potential clinical significance in early NSCLC. These results also suggest that different metabolic parameters should be selected to evaluate the prognosis of patients with different tumor stages.

Metabolism is a flexible system that maintains tissue growth and balance, tumor cells in the process of development respond to the external environment and can change their own metabolism, and metabolic changes are rarely limited to the tumor itself. Therefore, to understand the metabolism of cancer, it is necessary to study how the metabolic phenotype evolves over time and how tumor affect the metabolic changes of patients (26). Many studies have suggested that tumor can affect the metabolism of other organs in different ways (8, 27). Combined with our research results, when the tumor size was less than 4 cm, the prediction efficiency of the tumor metabolism level was the highest, which may be because other organs were not greatly affected by the tumor. When the tumor size reached 4-7 cm, the liver glucose metabolism level was significantly related to DFS, and combining the tumor metabolism and organ metabolism level can significantly increase the predictive efficiency. This may also explain why some PET/CT tumor metabolism parameters can only be applied to certain tumor stages and cannot be used throughout the tumor development stage. Therefore, with the development of tumor, metabolic changes of other organs should also be included for comprehensive evaluation of patient prognosis, since tumor regulate liver metabolism mainly through inflammatory factors such as IL-6 (8, 9), Linking changes in the metabolism of distal organs with clinical treatment methods may be beneficial in clinical practice for the treatment of cancer patients.

With continuous in-depth tumor research, related studies have found that tumor can inhibit the function of immunocytes through glycolysis metabolites (28, 29). This also makes it possible to combine PET glucose metabolism parameters with tumor immunity. Many studies have applied PET metabolic parameters to predict the effect of immunotherapy (30, 31). The results of our study show that there is no significant change in the metabolic level of the spleen, but related studies have confirmed its good prognostic ability (32–34). This finding also highlights the importance of the metabolism of various organs in tumor treatment. In addition to immunotherapy, PET metabolic parameters also have good performance in predicting the efficacy of traditional chemotherapy (35, 36). A recent study showed that patients with NSCLC whose tumor size is 4-7 cm are more likely to benefit from neoadjuvant chemotherapy (37). Combined with the results of our study, we speculate that the level of glucose metabolism in other organs begins to affect the DFS of patients when the tumor size is 4-7 cm. Therefore, is there a relationship between the effect of tumor on glucose metabolism in other organs and whether patients benefit from chemotherapy? This is a question worth discussing. In addition to glucose metabolism, other metabolic pathways play an important role in the development of tumor. A recent study suggests that glutamine may be more important to tumor cells than glucose (38); therefore, the application of PET tracers for glutamine will allow us to better understand the metabolic changes of tumor (39, 40).

This study still has some limitations. First, this is a retrospective single-centre study. Further prospective, multicentre, and animal experiment studies should be conducted to verify our findings. Moreover, we did not record the smoking status and lung function of the patients or whether there were specific complications that may affect the DFS of the patients. Because the patients only underwent PET/CT examination one time before the operation, we could not observe the changes in the metabolism of various organs at different stages of the tumor in the same patient. Second, not all patients underwent whole-body PET/CT scans, so we could not investigate the metabolic changes of other organs, such as the kidney. We only studied the changes in glucose metabolism, and other metabolic patterns should also be further studied.

## Conclusion

Our study found that in patients with early NSCLC, tumor glucose metabolism gradually increases with tumor size and distally regulates the liver and reduces liver metabolism in the primary lesion area, is associated with disease-free survival. When the tumor size is 4-7cm, combining the metabolic levels of remote organs such as the liver and spleen can better predict the prognosis of the patients with NSCLC.

## Data Availability Statement

The raw data supporting the conclusions of this article will be made available by the authors, without undue reservation.

## Ethics Statement

The studies involving human participants were reviewed and approved by Ethics Committee of the Third Xiangya Hospital. Written informed consent for participation was not required for this study in accordance with the national legislation and the institutional requirements.

## Author Contributions

HT and MM contributed equally to the literature research, drafting, interpretation, and writing of the manuscript. JH and WZ contributed to the statistics. SH contributed to the collection of patient data. KZ and PR contributed to the design of this work, image interpretation, and writing of the manuscript. All authors contributed to the article and approved the submitted version.

## Funding

This research is supported by the Wisdom Accumulation and Talent Cultivation Project of The Third Xiangya Hospital of Central South University.

## Conflict of Interest

The authors declare that the research was conducted in the absence of any commercial or financial relationships that could be construed as a potential conflict of interest.

## Publisher’s Note

All claims expressed in this article are solely those of the authors and do not necessarily represent those of their affiliated organizations, or those of the publisher, the editors and the reviewers. Any product that may be evaluated in this article, or claim that may be made by its manufacturer, is not guaranteed or endorsed by the publisher.

## Supplementary Material

Supplementary Figure S1 In the first group of patients, survival curves were drawn according to the SUVmax level **(A)**, TLR **(C)**, and TSR **(E)**. According to the distribution map, the best cut-off values were 4.6 for SUVmax **(B)**, 1.92 for TLR **(D)** and 2.57 for TSR **(F)**.

Supplementary Figure S2 In the second group of patients, survival curves were drawn according to the SUVmax level **(A)** and TLR **(C)**. According to the distribution map, the best cut-off values were 7.69 for SUVmax **(B)** and 3.6 for TLR **(D)**. In tumor patients with a tumor size of 4-5 cm, the survival curve based on liver SUVmean is shown in **(E)**, and the optimal cut-off value was 2.15 **(F)**.

Supplementary Figure S3 In patients with a tumor size of 5-7 cm, survival curves were drawn according to the liver SUVmean **(A)**, TLR **(C)**, and TSR **(E)**. According to the distribution map, the best cut-off values were 1.95 for liver SUVmean **(B)**, 5.89 for TLR **(D)** and 6.32 for TSR **(F)**.
