# Supplementary figures and images for: Glucose Metabolism Reprogramming of Primary Tumor and the Liver Is Associated With Disease-Free Survival in Patients With Early NSCLC
Source: Front Oncol. 2021 Oct 28;11:752036. doi: 10.3389/fonc.2021.752036 (PMC8581354; doi:10.3389/fonc.2021.752036)

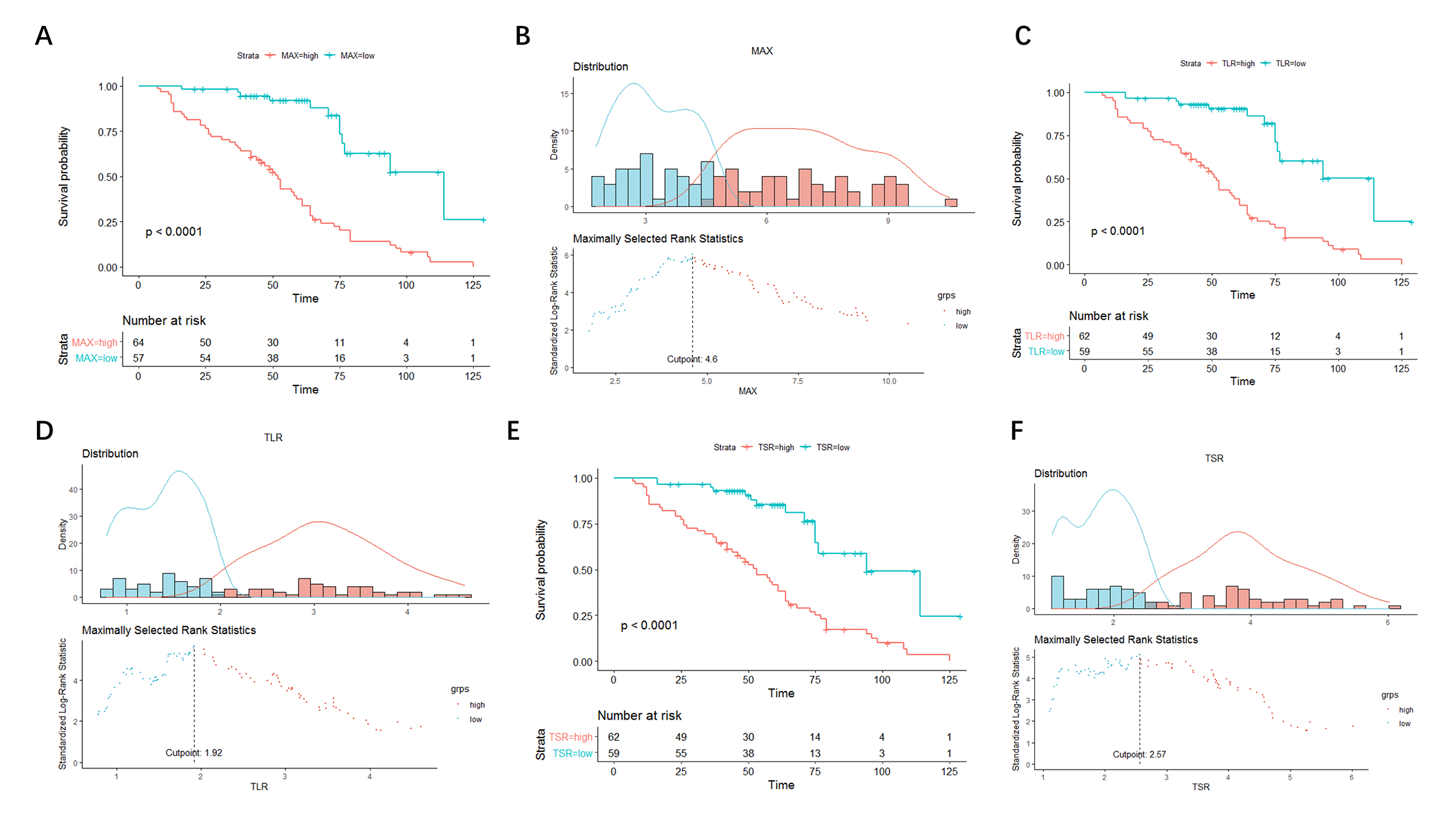

Supplement: Supplementary Figure S1 — In the first group of patients, survival curves were drawn according to the SUVmax level (A), TLR (C), and TSR (E). According to the distribution map, the best cut-off values were 4.6 for SUVmax (B), 1.92 for TLR (D) and 2.57 for TSR (F). [file Image_1.tif]

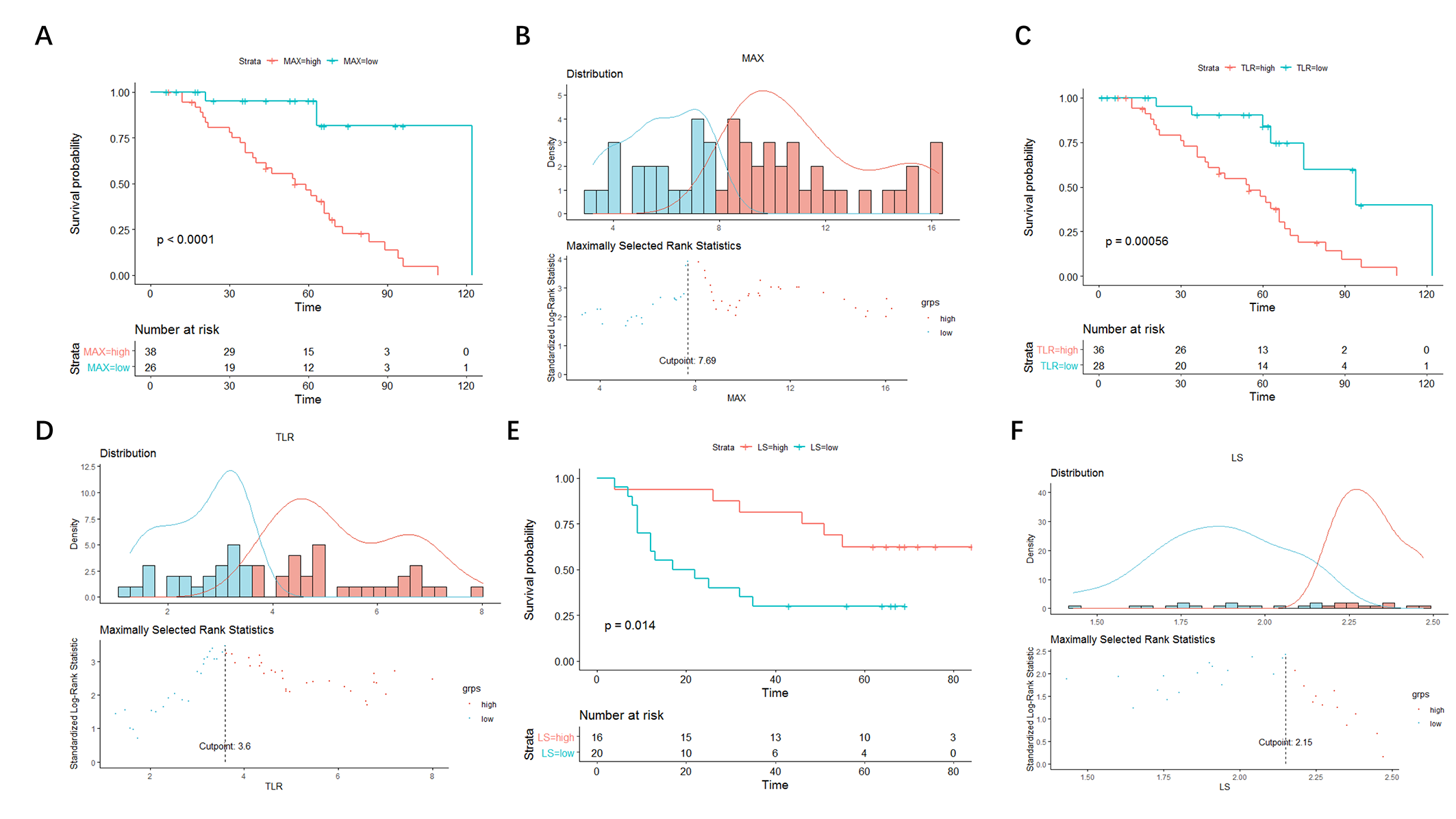

Supplement: Supplementary Figure S2 — In the second group of patients, survival curves were drawn according to the SUVmax level (A) and TLR (C). According to the distribution map, the best cut-off values were 7.69 for SUVmax (B) and 3.6 for TLR (D). In tumor patients with a tumor size of 4-5 cm, the survival curve based on liver SUVmean is shown in (E), and the optimal cut-off value was 2.15 (F). [file Image_2.tif]

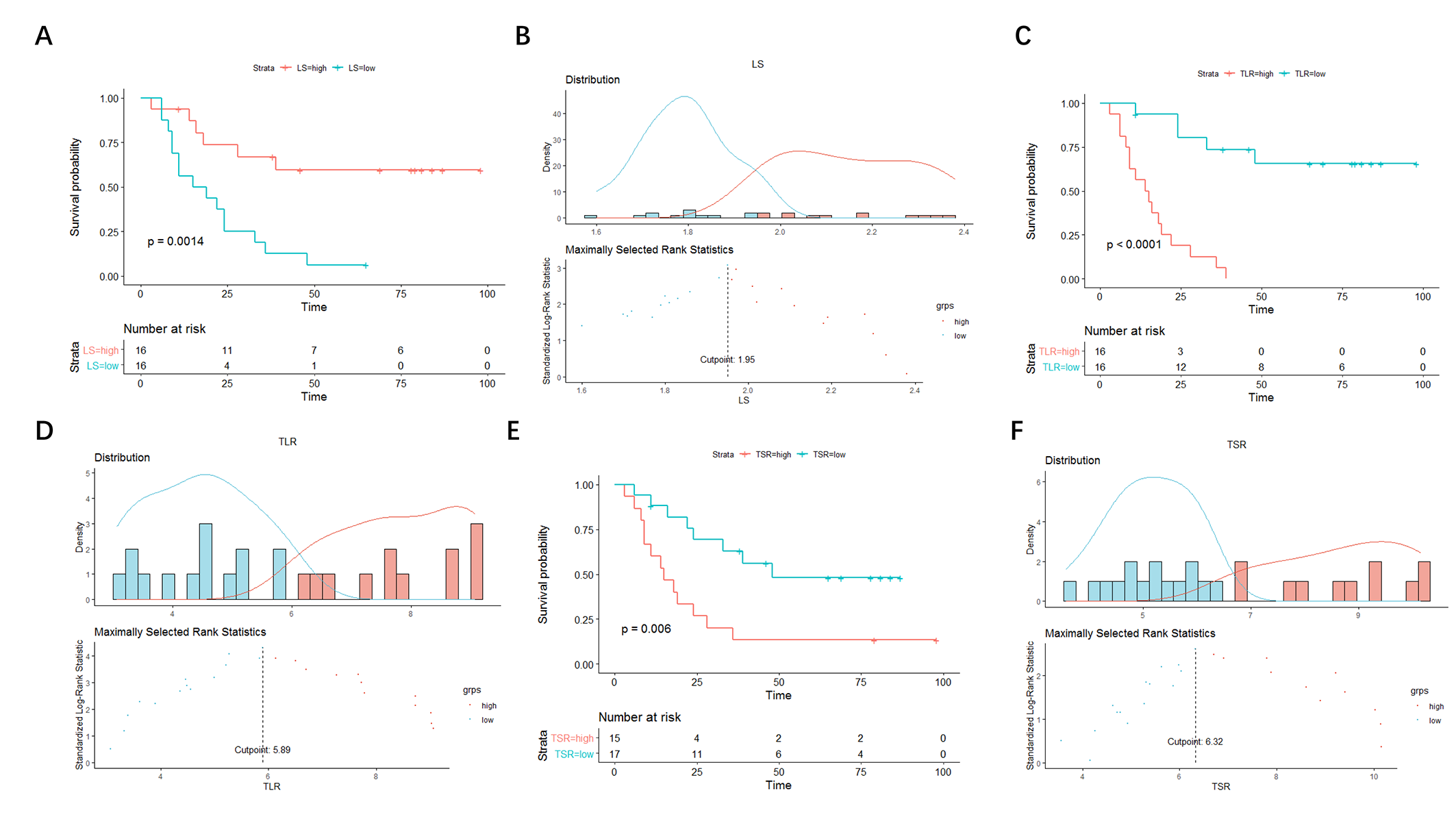

Supplement: Supplementary Figure S3 — In patients with a tumor size of 5-7 cm, survival curves were drawn according to the liver SUVmean (A), TLR (C), and TSR (E). According to the distribution map, the best cut-off values were 1.95 for liver SUVmean (B), 5.89 for TLR (D) and 6.32 for TSR (F). [file Image_3.tif]
